# *N*-(1,3-Benzo­thia­zol-2-yl)acetamide

**DOI:** 10.1107/S160053681302730X

**Published:** 2013-10-09

**Authors:** Prakash S Nayak, B. Narayana, Jerry P. Jasinski, H. S. Yathirajan, Manpreet Kaur

**Affiliations:** a Department of Studies in Chemistry, Mangalore University, Mangalagangotri 574 199, India; b Department of Chemistry, Keene State College, 229 Main Street, Keene, NH 03435-2001, USA; c Department of Studies in Chemistry, University of Mysore, Manasagangotri, Mysore 570 006, India

## Abstract

The title compound, C_9_H_8_N_2_OS, crystallizes with two mol­ecules (*A* and *B*) in the asymmetric unit. The dihedral angles between the mean planes of the 1,3-benzo­thia­zol-2-yl ring system and the acetamide group are 2.7 (4) (mol­ecule *A*) and 7.2 (2) Å (mol­ecule *B*). In the crystal, pairs of N—H⋯N hydrogen bonds link the *A* and *B* mol­ecules into dimers, generating *R*_2_^2^(8) loops. The dimers stack along [100].

## Related literature

For the related crystal structure of the acetamide derivatives, see: Jasinski *et al.* (2013 ▶); Fun *et al.* (2011*a* ▶,*b* ▶, 2012 ▶).
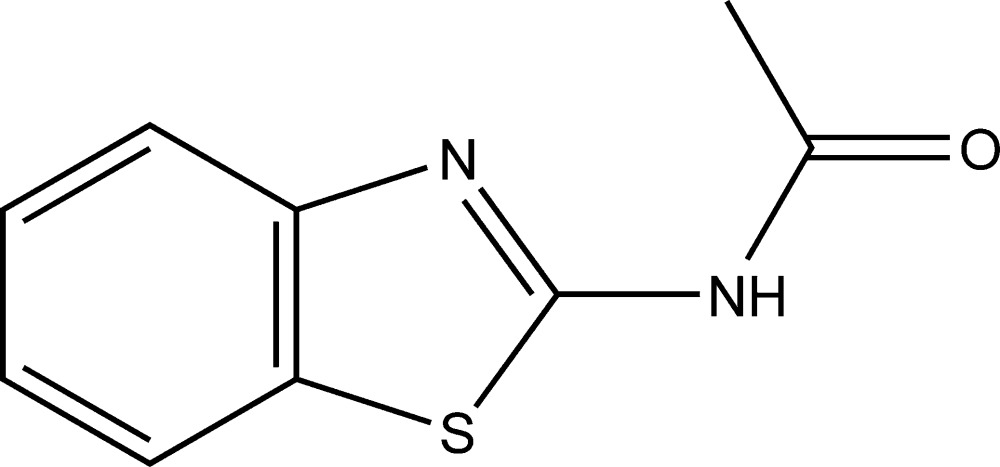


## Experimental

### 

#### Crystal data

C_9_H_8_N_2_OS
*M* *_r_* = 192.24
Monoclinic, 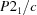

*a* = 11.1852 (4) Å
*b* = 7.4037 (4) Å
*c* = 20.9189 (8) Å
β = 94.408 (3)°
*V* = 1727.21 (13) Å^3^
*Z* = 8
Mo *K*α radiation
μ = 0.33 mm^−1^
*T* = 173 K
0.45 × 0.24 × 0.15 mm

#### Data collection

Agilent Xcalibur (Eos, Gemini) diffractometer
Absorption correction: multi-scan (*CrysAlis PRO* and *CrysAlis RED*; Agilent, 2012 ▶)*T*_min_ = 0.770, *T*_max_ = 1.000
20845 measured reflections
5918 independent reflections
4622 reflections with *I* > 2σ(*I*)
*R*_int_ = 0.033

#### Refinement

*R*[*F* ^2^ > 2σ(*F* ^2^)] = 0.044
*wR*(*F* ^2^) = 0.109
*S* = 1.08
5918 reflections
237 parameters
H-atom parameters constrained
Δρ_max_ = 0.44 e Å^−3^
Δρ_min_ = −0.26 e Å^−3^

### 

Data collection: *CrysAlis PRO* (Agilent, 2012 ▶); cell refinement: *CrysAlis PRO*; data reduction: *CrysAlis RED* (Agilent, 2012 ▶); program(s) used to solve structure: *SUPERFLIP* (Palatinus & Chapuis, 2007 ▶); program(s) used to refine structure: *SHELXL2013* (Sheldrick, 2008 ▶); molecular graphics: *OLEX2* (Dolomanov *et al.*, 2009 ▶); software used to prepare material for publication: *OLEX2*.

## Supplementary Material

Crystal structure: contains datablock(s) I. DOI: 10.1107/S160053681302730X/hb7144sup1.cif

Structure factors: contains datablock(s) I. DOI: 10.1107/S160053681302730X/hb7144Isup2.hkl

Supplementary material file. DOI: 10.1107/S160053681302730X/hb7144Isup3.cml

Additional supplementary materials:  crystallographic information; 3D view; checkCIF report

## Figures and Tables

**Table 1:** Hydrogen-bond geometry (Å, °)

*D*—H⋯*A*	*D*—H	H⋯*A*	*D*⋯*A*	*D*—H⋯*A*
N2*A*—H2*A*⋯N1*B*	0.86	2.11	2.9700 (16)	176
N2*B*—H2*B*⋯N1*A*	0.86	2.14	2.9749 (16)	165

**Fig. 1.**
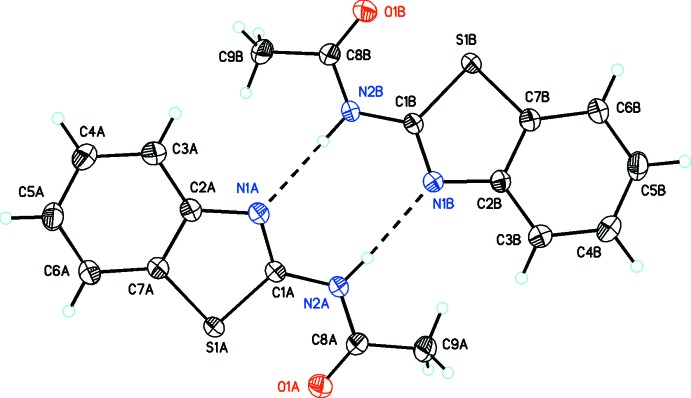
ORTEP drawing of (I) showing 50% probability displacement ellipsoids. Dashed lines indicate N—H···N intermolecular hydrogen bonds between A and B forming R22(8) graph set motifs.

**Fig. 2.**
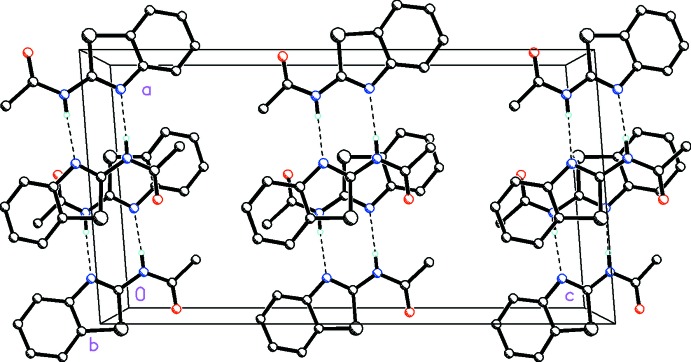
Molecular packing for (I) viewed along the b axis. Dashed lines indicate N—H···N intermolecular hydrogen bonds forming R22(8) graph set motifs which link the molecules into dimers along [100]. H atoms not involved in hydrogen bonding have been removed for clarity.

**Fig. 3.**
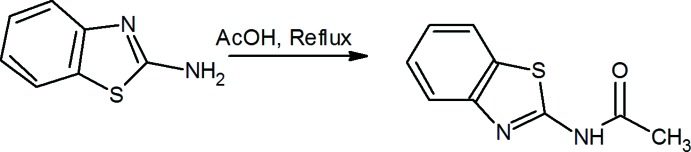
Synthesis scheme for (I).

## supplementary crystallographic information

### 1. Comment

In continuation of our work on the synthesis of acetamide derivatives (Jasinski *et al.* 2013), we report herein the crystal structure of the title compound, C_9_H_8_N_2_OS, (I). Some of the related crystal structures of similar acetamide derivatives include, N-(3-chloro-4-fluorophenyl)acetamide, N-(4-bromophenyl)-2-(naphthalen-1- yl)acetamide and N-(3,5-dichlorophenyl)-2-(naphthalen-1-yl)acetamide (Fun *et al.* 2011*a*,*b*, 2012).

The title compound, (I) crystallizes with two independent molecules (A & B) in the asymmetric unit (Fig.1). The dihedral angle between the mean planes of the 1,3-benzothiazol-2-yl ring and the acetamide group is 2.7 (4)° (A) and 7.2 (2)Å (B),(Fig. 2). In the crystal, N—H···N hydrogen bonds forming R_2_^2^(8) graph set motifs which link the molecules into dimers, which stack along [100].

### 2. Experimental

2-Aminobenzothiazole (1 mmol) were dissolved in a 30 ml acetic acid and it was refluxed for 3 hrs (Fig.3). The reaction mixture was cooled and poured into ice cold water. The precipitate obtained was obtained by filtration and recrystallized in ethanol. Colorless blocks were grown from methanol solution by the slow evaporation method and was used as such for X-ray studies (M.P.: 453-455 K).

### 3. Refinement

All of the H atoms were placed in their calculated positions and then refined using the riding model with Atom—H lengths of 0.93Å (CH), 0.96Å (CH_3_) or 0.86Å (NH). Isotropic displacement parameters for these atoms were set to 1.2 (CH, NH) or 1.5 (CH_3_) times *U*_eq_ of the parent atom. Idealised methyl were refined as rotating groups.

### Crystal data

**Table:**

C_9_H_8_N_2_OS	*D*_x_ = 1.479 Mg m^−^^3^
*M**_r_* = 192.24	Melting point: 453 K
Monoclinic, *P*2_1_/*c*	Mo *K*α radiation, λ = 0.71073 Å
*a* = 11.1852 (4) Å	Cell parameters from 5326 reflections
*b* = 7.4037 (4) Å	θ = 3.3–32.7°
*c* = 20.9189 (8) Å	µ = 0.33 mm^−^^1^
β = 94.408 (3)°	*T* = 173 K
*V* = 1727.21 (13) Å^3^	Block, colorless
*Z* = 8	0.45 × 0.24 × 0.15 mm
*F*(000) = 800	

### Data collection

**Table:**

Agilent Xcalibur (Eos, Gemini) diffractometer	5918 independent reflections
Radiation source: Enhance (Mo) X-ray Source	4622 reflections with *I* > 2σ(*I*)
Detector resolution: 16.0416 pixels mm^-1^	*R*_int_ = 0.033
ω scans	θ_max_ = 32.8°, θ_min_ = 3.3°
Absorption correction: multi-scan *CrysAlis PRO* and *CrysAlis RED*, Agilent (2012).	*h* = −16→16
*T*_min_ = 0.770, *T*_max_ = 1.000	*k* = −10→9
20845 measured reflections	*l* = −30→31

### Refinement

**Table:**

Refinement on *F*^2^	Primary atom site location: structure-invariant direct methods
Least-squares matrix: full	Hydrogen site location: inferred from neighbouring sites
*R*[*F*^2^ > 2σ(*F*^2^)] = 0.044	H-atom parameters constrained
*wR*(*F*^2^) = 0.109	*w* = 1/[σ^2^(*F*_o_^2^) + (0.045*P*)^2^ + 0.4973*P*] where *P* = (*F*_o_^2^ + 2*F*_c_^2^)/3
*S* = 1.08	(Δ/σ)_max_ = 0.001
5918 reflections	Δρ_max_ = 0.44 e Å^−^^3^
237 parameters	Δρ_min_ = −0.26 e Å^−^^3^
0 restraints	

### Special details

**Table:**

Geometry. All esds (except the esd in the dihedral angle between two l.s. planes) are estimated using the full covariance matrix. The cell esds are taken into account individually in the estimation of esds in distances, angles and torsion angles; correlations between esds in cell parameters are only used when they are defined by crystal symmetry. An approximate (isotropic) treatment of cell esds is used for estimating esds involving l.s. planes.

### Fractional atomic coordinates and isotropic or equivalent isotropic displacement parameters (Å^2^)

**Table:**

	*x*	*y*	*z*	*U*_iso_*/*U*_eq_	
S1A	0.38148 (3)	0.72688 (5)	0.50197 (2)	0.02464 (9)	
O1A	0.45185 (9)	0.64286 (18)	0.62295 (5)	0.0349 (3)	
N1A	0.58652 (10)	0.79642 (18)	0.45587 (5)	0.0242 (2)	
N2A	0.60552 (10)	0.72112 (17)	0.56435 (5)	0.0232 (2)	
H2A	0.6815	0.7393	0.5650	0.028*	
C1A	0.53676 (11)	0.75086 (19)	0.50776 (6)	0.0208 (3)	
C2A	0.49856 (11)	0.8192 (2)	0.40579 (6)	0.0228 (3)	
C3A	0.51887 (13)	0.8704 (2)	0.34317 (7)	0.0301 (3)	
H3A	0.5961	0.8949	0.3320	0.036*	
C4A	0.42227 (14)	0.8841 (2)	0.29819 (7)	0.0315 (3)	
H4A	0.4349	0.9165	0.2563	0.038*	
C5A	0.30593 (13)	0.8500 (2)	0.31478 (7)	0.0303 (3)	
H5A	0.2423	0.8596	0.2837	0.036*	
C6A	0.28360 (13)	0.8024 (2)	0.37647 (7)	0.0284 (3)	
H6A	0.2059	0.7813	0.3876	0.034*	
C7A	0.38110 (12)	0.7869 (2)	0.42159 (6)	0.0225 (3)	
C8A	0.55898 (12)	0.6640 (2)	0.61966 (6)	0.0241 (3)	
C9A	0.64887 (14)	0.6310 (2)	0.67511 (7)	0.0312 (3)	
H9AA	0.7267	0.6141	0.6597	0.047*	
H9AB	0.6268	0.5247	0.6977	0.047*	
H9AC	0.6508	0.7329	0.7035	0.047*	
S1B	1.06929 (3)	0.76516 (5)	0.50481 (2)	0.02415 (9)	
O1B	0.99661 (9)	0.63954 (18)	0.38920 (5)	0.0338 (3)	
N1B	0.86730 (10)	0.77933 (17)	0.55907 (5)	0.0230 (2)	
N2B	0.84393 (10)	0.70106 (17)	0.45051 (5)	0.0232 (2)	
H2B	0.7673	0.7064	0.4517	0.028*	
C1B	0.91433 (11)	0.74614 (19)	0.50500 (6)	0.0200 (2)	
C2B	0.95787 (11)	0.8261 (2)	0.60567 (6)	0.0210 (3)	
C3B	0.94173 (13)	0.8677 (2)	0.66959 (7)	0.0288 (3)	
H3B	0.8654	0.8664	0.6844	0.035*	
C4B	1.04033 (14)	0.9107 (2)	0.71058 (7)	0.0316 (3)	
H4B	1.0302	0.9376	0.7533	0.038*	
C5B	1.15469 (13)	0.9140 (2)	0.68865 (7)	0.0303 (3)	
H5B	1.2198	0.9446	0.7169	0.036*	
C6B	1.17329 (12)	0.8727 (2)	0.62580 (7)	0.0273 (3)	
H6B	1.2499	0.8745	0.6114	0.033*	
C7B	1.07368 (11)	0.8283 (2)	0.58475 (6)	0.0219 (3)	
C8B	0.88922 (12)	0.6480 (2)	0.39429 (6)	0.0242 (3)	
C9B	0.79789 (13)	0.5998 (2)	0.34107 (7)	0.0300 (3)	
H9BA	0.7214	0.5827	0.3581	0.045*	
H9BB	0.8214	0.4902	0.3210	0.045*	
H9BC	0.7922	0.6954	0.3100	0.045*	

### Atomic displacement parameters (Å^2^)

**Table:**

	*U*^11^	*U*^22^	*U*^33^	*U*^12^	*U*^13^	*U*^23^
S1A	0.01604 (15)	0.0370 (2)	0.02094 (15)	−0.00082 (13)	0.00206 (11)	0.00004 (13)
O1A	0.0235 (5)	0.0539 (8)	0.0277 (5)	−0.0055 (5)	0.0038 (4)	0.0070 (5)
N1A	0.0172 (5)	0.0334 (7)	0.0218 (5)	−0.0006 (4)	0.0010 (4)	0.0034 (5)
N2A	0.0165 (5)	0.0327 (7)	0.0203 (5)	−0.0008 (4)	0.0007 (4)	0.0012 (5)
C1A	0.0166 (5)	0.0248 (7)	0.0211 (6)	0.0002 (5)	0.0012 (4)	−0.0005 (5)
C2A	0.0187 (6)	0.0268 (7)	0.0226 (6)	0.0007 (5)	0.0003 (5)	0.0013 (5)
C3A	0.0244 (7)	0.0408 (9)	0.0253 (7)	0.0003 (6)	0.0038 (5)	0.0061 (6)
C4A	0.0329 (8)	0.0394 (9)	0.0222 (6)	0.0038 (6)	0.0014 (5)	0.0054 (6)
C5A	0.0278 (7)	0.0382 (9)	0.0239 (6)	0.0065 (6)	−0.0045 (5)	−0.0010 (6)
C6A	0.0201 (6)	0.0391 (9)	0.0256 (6)	0.0033 (6)	−0.0010 (5)	−0.0024 (6)
C7A	0.0192 (6)	0.0273 (7)	0.0210 (6)	0.0017 (5)	0.0015 (4)	−0.0012 (5)
C8A	0.0243 (6)	0.0273 (7)	0.0206 (6)	−0.0008 (5)	0.0015 (5)	−0.0001 (5)
C9A	0.0303 (7)	0.0387 (9)	0.0240 (7)	0.0007 (6)	−0.0016 (5)	0.0046 (6)
S1B	0.01533 (15)	0.0370 (2)	0.02013 (15)	−0.00067 (12)	0.00165 (11)	−0.00226 (13)
O1B	0.0229 (5)	0.0521 (8)	0.0269 (5)	−0.0017 (5)	0.0051 (4)	−0.0072 (5)
N1B	0.0174 (5)	0.0326 (7)	0.0190 (5)	0.0015 (4)	0.0010 (4)	−0.0006 (4)
N2B	0.0154 (5)	0.0346 (7)	0.0193 (5)	−0.0008 (4)	−0.0010 (4)	−0.0007 (5)
C1B	0.0154 (5)	0.0247 (7)	0.0199 (5)	0.0008 (4)	0.0002 (4)	0.0008 (5)
C2B	0.0181 (6)	0.0251 (7)	0.0194 (6)	0.0021 (5)	−0.0005 (4)	0.0006 (5)
C3B	0.0244 (7)	0.0398 (9)	0.0223 (6)	0.0002 (6)	0.0030 (5)	−0.0037 (6)
C4B	0.0332 (8)	0.0413 (9)	0.0200 (6)	−0.0007 (6)	−0.0002 (5)	−0.0040 (6)
C5B	0.0277 (7)	0.0366 (9)	0.0252 (7)	−0.0039 (6)	−0.0065 (5)	−0.0018 (6)
C6B	0.0194 (6)	0.0369 (9)	0.0249 (6)	−0.0025 (5)	−0.0020 (5)	−0.0010 (6)
C7B	0.0191 (6)	0.0255 (7)	0.0209 (6)	0.0006 (5)	0.0001 (4)	0.0006 (5)
C8B	0.0226 (6)	0.0297 (8)	0.0203 (6)	−0.0026 (5)	0.0010 (5)	−0.0004 (5)
C9B	0.0310 (7)	0.0377 (9)	0.0208 (6)	−0.0051 (6)	−0.0014 (5)	−0.0038 (6)

### Geometric parameters (Å, º)

**Table:**

S1A—C1A	1.7407 (13)	S1B—C1B	1.7392 (13)
S1A—C7A	1.7390 (14)	S1B—C7B	1.7333 (13)
O1A—C8A	1.2156 (17)	O1B—C8B	1.2157 (16)
N1A—C1A	1.3018 (17)	N1B—C1B	1.3068 (16)
N1A—C2A	1.3913 (17)	N1B—C2B	1.3945 (17)
N2A—H2A	0.8600	N2B—H2B	0.8600
N2A—C1A	1.3791 (17)	N2B—C1B	1.3757 (16)
N2A—C8A	1.3715 (17)	N2B—C8B	1.3733 (17)
C2A—C3A	1.3989 (19)	C2B—C3B	1.3974 (18)
C2A—C7A	1.3997 (18)	C2B—C7B	1.3988 (18)
C3A—H3A	0.9300	C3B—H3B	0.9300
C3A—C4A	1.381 (2)	C3B—C4B	1.381 (2)
C4A—H4A	0.9300	C4B—H4B	0.9300
C4A—C5A	1.395 (2)	C4B—C5B	1.392 (2)
C5A—H5A	0.9300	C5B—H5B	0.9300
C5A—C6A	1.379 (2)	C5B—C6B	1.381 (2)
C6A—H6A	0.9300	C6B—H6B	0.9300
C6A—C7A	1.3910 (19)	C6B—C7B	1.3928 (18)
C8A—C9A	1.4957 (19)	C8B—C9B	1.4952 (19)
C9A—H9AA	0.9600	C9B—H9BA	0.9600
C9A—H9AB	0.9600	C9B—H9BB	0.9600
C9A—H9AC	0.9600	C9B—H9BC	0.9600
			
C7A—S1A—C1A	88.25 (6)	C7B—S1B—C1B	88.51 (6)
C1A—N1A—C2A	109.66 (11)	C1B—N1B—C2B	109.42 (11)
C1A—N2A—H2A	118.3	C1B—N2B—H2B	118.2
C8A—N2A—H2A	118.3	C8B—N2B—H2B	118.2
C8A—N2A—C1A	123.41 (12)	C8B—N2B—C1B	123.62 (11)
N1A—C1A—S1A	117.17 (10)	N1B—C1B—S1B	117.01 (10)
N1A—C1A—N2A	120.74 (12)	N1B—C1B—N2B	121.32 (12)
N2A—C1A—S1A	122.08 (10)	N2B—C1B—S1B	121.66 (10)
N1A—C2A—C3A	125.57 (12)	N1B—C2B—C3B	125.69 (12)
N1A—C2A—C7A	115.07 (12)	N1B—C2B—C7B	115.13 (11)
C3A—C2A—C7A	119.36 (12)	C3B—C2B—C7B	119.18 (12)
C2A—C3A—H3A	120.6	C2B—C3B—H3B	120.4
C4A—C3A—C2A	118.89 (13)	C4B—C3B—C2B	119.29 (13)
C4A—C3A—H3A	120.6	C4B—C3B—H3B	120.4
C3A—C4A—H4A	119.6	C3B—C4B—H4B	119.7
C3A—C4A—C5A	120.87 (13)	C3B—C4B—C5B	120.63 (13)
C5A—C4A—H4A	119.6	C5B—C4B—H4B	119.7
C4A—C5A—H5A	119.4	C4B—C5B—H5B	119.4
C6A—C5A—C4A	121.23 (13)	C6B—C5B—C4B	121.29 (13)
C6A—C5A—H5A	119.4	C6B—C5B—H5B	119.4
C5A—C6A—H6A	121.1	C5B—C6B—H6B	121.1
C5A—C6A—C7A	117.84 (13)	C5B—C6B—C7B	117.86 (13)
C7A—C6A—H6A	121.1	C7B—C6B—H6B	121.1
C2A—C7A—S1A	109.84 (10)	C2B—C7B—S1B	109.90 (10)
C6A—C7A—S1A	128.36 (11)	C6B—C7B—S1B	128.35 (10)
C6A—C7A—C2A	121.80 (12)	C6B—C7B—C2B	121.74 (12)
O1A—C8A—N2A	121.82 (13)	O1B—C8B—N2B	121.45 (12)
O1A—C8A—C9A	122.81 (13)	O1B—C8B—C9B	123.07 (13)
N2A—C8A—C9A	115.37 (12)	N2B—C8B—C9B	115.48 (12)
C8A—C9A—H9AA	109.5	C8B—C9B—H9BA	109.5
C8A—C9A—H9AB	109.5	C8B—C9B—H9BB	109.5
C8A—C9A—H9AC	109.5	C8B—C9B—H9BC	109.5
H9AA—C9A—H9AB	109.5	H9BA—C9B—H9BB	109.5
H9AA—C9A—H9AC	109.5	H9BA—C9B—H9BC	109.5
H9AB—C9A—H9AC	109.5	H9BB—C9B—H9BC	109.5
			
N1A—C2A—C3A—C4A	178.73 (15)	N1B—C2B—C3B—C4B	179.62 (15)
N1A—C2A—C7A—S1A	0.23 (17)	N1B—C2B—C7B—S1B	−1.33 (16)
N1A—C2A—C7A—C6A	−179.33 (14)	N1B—C2B—C7B—C6B	179.88 (14)
C1A—S1A—C7A—C2A	0.29 (11)	C1B—S1B—C7B—C2B	1.31 (11)
C1A—S1A—C7A—C6A	179.81 (15)	C1B—S1B—C7B—C6B	180.00 (15)
C1A—N1A—C2A—C3A	179.14 (15)	C1B—N1B—C2B—C3B	−178.89 (15)
C1A—N1A—C2A—C7A	−0.81 (19)	C1B—N1B—C2B—C7B	0.50 (18)
C1A—N2A—C8A—O1A	−3.1 (2)	C1B—N2B—C8B—O1B	−0.8 (2)
C1A—N2A—C8A—C9A	177.18 (14)	C1B—N2B—C8B—C9B	178.36 (13)
C2A—N1A—C1A—S1A	1.07 (16)	C2B—N1B—C1B—S1B	0.60 (16)
C2A—N1A—C1A—N2A	−179.72 (13)	C2B—N1B—C1B—N2B	−178.39 (13)
C2A—C3A—C4A—C5A	0.9 (3)	C2B—C3B—C4B—C5B	0.4 (3)
C3A—C2A—C7A—S1A	−179.73 (12)	C3B—C2B—C7B—S1B	178.10 (12)
C3A—C2A—C7A—C6A	0.7 (2)	C3B—C2B—C7B—C6B	−0.7 (2)
C3A—C4A—C5A—C6A	0.3 (3)	C3B—C4B—C5B—C6B	−0.7 (3)
C4A—C5A—C6A—C7A	−0.9 (3)	C4B—C5B—C6B—C7B	0.3 (2)
C5A—C6A—C7A—S1A	−179.09 (13)	C5B—C6B—C7B—S1B	−178.14 (13)
C5A—C6A—C7A—C2A	0.4 (2)	C5B—C6B—C7B—C2B	0.4 (2)
C7A—S1A—C1A—N1A	−0.82 (12)	C7B—S1B—C1B—N1B	−1.16 (12)
C7A—S1A—C1A—N2A	179.98 (13)	C7B—S1B—C1B—N2B	177.82 (12)
C7A—C2A—C3A—C4A	−1.3 (2)	C7B—C2B—C3B—C4B	0.2 (2)
C8A—N2A—C1A—S1A	2.6 (2)	C8B—N2B—C1B—S1B	7.6 (2)
C8A—N2A—C1A—N1A	−176.56 (14)	C8B—N2B—C1B—N1B	−173.46 (14)

### Hydrogen-bond geometry (Å, º)

**Table:**

*D*—H···*A*	*D*—H	H···*A*	*D*···*A*	*D*—H···*A*
N2*A*—H2*A*···N1*B*	0.86	2.11	2.9700 (16)	176
N2*B*—H2*B*···N1*A*	0.86	2.14	2.9749 (16)	165

## References

[bb1] Agilent (2012). *CrysAlis PRO* and *CrysAlis RED* Agilent Technologies, Yarnton, Oxfordshire, England.

[bb2] Dolomanov, O. V., Bourhis, L. J., Gildea, R. J., Howard, J. A. K. & Puschmann, H. (2009). *J. Appl. Cryst.* **42**, 339–341.

[bb3] Fun, H.-K., Loh, W.-S., Shetty, D. N., Narayana, B. & Sarojini, B. K. (2012). *Acta Cryst.* E**68**, o1348. 10.1107/S160053681201416X PMC3344482 22590244

[bb4] Fun, H.-K., Quah, C. K., Narayana, B., Nayak, P. S. & Sarojini, B. K. (2011*a*). *Acta Cryst.* E**67**, o2926–o2927. 10.1107/S1600536811041110 PMC3247340 22219958

[bb5] Fun, H.-K., Quah, C. K., Narayana, B., Nayak, P. S. & Sarojini, B. K. (2011*b*). *Acta Cryst.* E**67**, o2941–o2942. 10.1107/S1600536811041468 PMC3247353 22219971

[bb6] Jasinski, J. P., Guild, C. J., Yathirajan, H. S., Narayana, B. & Samshuddin, S. (2013). *Acta Cryst.* E**69**, o461. 10.1107/S1600536813005448 PMC3588444 23476627

[bb7] Palatinus, L. & Chapuis, G. (2007). *J. Appl. Cryst.* **40**, 786–790.

[bb8] Sheldrick, G. M. (2008). *Acta Cryst.* A**64**, 112–122. 10.1107/S0108767307043930 18156677

